# Evaluation of ambulance calls for patients over 65 years of age in İzmir, Turkey: a two-year retrospective analysis

**DOI:** 10.3906/sag-2010-152

**Published:** 2021-06-28

**Authors:** Ahu PAKDEMİRLİ, Başak BAYRAM, Erkan GÜVENÇ, Hülya ELLİDOKUZ

**Affiliations:** 1 Department of Physiology, Gülhane School of Medicine, University of Health Sciences, Ankara Turkey; 2 Department of Emergency Medicine, Dokuz Eylül University, İzmir Turkey; 3 Department of Emergency Medicine, Buca Seyfi Demirsoy State Hospital, İzmir Turkey; 4 Department of Preventative Oncology, Dokuz Eylül University, İzmir Turkey

**Keywords:** Ambulance, aged, emergency medical services, geriatrics

## Abstract

**Background/aim:**

Analysis of interventions for special patient groups is important for the planning of health services, especially emergency medical services. In this study, we aimed to evaluate emergency medical service (EMS) interventions for the elderly and determine the decisive factors affecting transfer to the hospital of EMS team over 2 years (2017 and 2018) in İzmir.

**Materials and methods:**

Records of 112 emergency calls that were made between 2017 and 2018 followed up with interventions for patients aged 65 years and older were obtained from the 112 system. The reasons for the calls, outcomes, possible diagnoses of the patients, differences in time intervals and seasons, characteristics of the patients transferred to the hospital, and factors affecting the need for transfer to the hospital were investigated.

**Results:**

A total of 176,104 elderly patients with a mean age of 78.02 ± 8.0 years required ambulance services, and out of them, 66% were transferred to the hospital. Transfer to the hospital was significantly associated with the event location, sex, time interval, international classification of diseases (ICD) codes, and physical examination findings.

**Conclusion:**

Ambulance interventions are more frequently required in urban areas than in the countryside, and calls are mostly made during daytime hours and during winter months. The decision to transfer a patient to the hospital is based on the patient’s respiratory status, skin examination, state of consciousness, pulse, systolic blood pressure, call time, and the preliminary diagnosis of the crew.

## 1. Introduction

In the USA, elderly patients account for more than 15% of all emergency department (ED) patients National Center for Health Statistics. National Hospital Ambulatory Medical Care Survey (2014). Emergency Department Summary Tables. Atlanta, GA: Centers for Disease Control and Prevention; [online] Website https://www.cdc.gov/nchs/data/nhamcs/web_tables/2014_ed_web_tables.pdf [accessed 10.1.2020].. However, in Turkey, 20% of patients attending the ED are patients aged 65 years and older [1,2], and 34.2% of elderly patients arrive at the ED by ambulance [3]. In particular, the number of patients aged 80 years and older who are transferred by ambulance is higher than that of all other age groups [4]. Furthermore, when all patients transported by ambulance were evaluated, advanced age was reported as an independent risk factor for death in the next 7 days [5].

İzmir is the third most populous city in Turkey. In İzmir, people aged 65 years and older constitute 10.5% of the city’s entire population and the dependency rate in the elderly was reported to be 14.9% in 2017 [2]. According to a study evaluating ambulance interventions in İzmir in 2004−2005, the age group that most frequently required ambulance interventions was over 65 years, and the frequency was 3.7 times higher than that in other age groups [6]. In 2005, it was reported that 68% of all ambulance calls were made for patients over the age of 65 years, and 60.8% of these patients were hospitalized [7].

While people aged 65 years and older in Turkey constituted 8.0% of the country’s entire population in 2014, this number increased by 21.4% in the next 5 years İstatistiklerle Yaşlılar, 2019. Türkiye İstatistik Kurumu, Haber Bülteni (2020). Sayı: 33712 [online] https://tuikweb.tuik.gov.tr/PreHaberBultenleri.do?id=33712 [accessed 04.12.2020] (in Turkish).. This may have caused changes in the number of patients treated using ambulance services. On the other hand, in our literature research, we have not found any study that examined large patient series and that could be used to plan emergency medical services (EMS) in a region. Analysis of interventions for special patient groups is important for planning for all health services, and not only EMS. Given that the highest increase in ambulance use has been noted in elderly patients and patients with severe disease, alternative care models should be developed in EMS for this patient group [4]. Therefore, in this study, we aimed to evaluate EMS interventions for patients over 65 years of age and to determine the decisive factors affecting transfer to the hospital of EMS team, over 2 years (2017 and 2018) in İzmir.

## 2. Material and methods

This retrospective cross-sectional study was conducted with the approval of the Non-Interventional Ethics Committee of Dokuz Eylül University, Faculty of Medicine with decision number: 2019/09-48 and date: 10.04.2019 and the Health Ethics Committee of the İzmir Provincial Directorate with decision number: 77597247-619 and date: 24.09.2019. 

### 2.1. Sampling and data collection 

There are 136 ambulances in 30 districts of İzmir that are connected to the Provincial Ambulance Service of the Ministry of Health. At least three personnel work as a team of emergency aid ambulances in Turkey, and at least one physician or a paramedic acts as the team leader. If one of these is not available, an emergency medical technician who has completed the training of modules determined by the Ministry of Health acts as a team leader.

Records of all emergency calls made to 112 that were followed up with interventions for patients aged 65 years and older in İzmir, between 1 January 2017 and 31 December 2018, were obtained from the 112 Emergency Operation Management System (ARMAKOM©) in an Excel XML file. The file included the date and time of the call, age and sex of the patient, the ICD diagnosis code, the mode of termination of the call, the hospitals to which they were transferred, the state of consciousness of the patients, pupil examination, respiratory status, skin examination, blood pressure, pulse, and systolic/diastolic blood pressure. 

In the evaluation of all ambulance interventions throughout the city, data deficiencies were not regarded. Records with missing data and records of transfers between hospitals, use of the ambulance as a precautionary measure, and use of ambulance services for transfer to home were excluded in the analysis of ICD codes. Only records of patients who were transported to the hospital or received the on-site intervention were included in the analysis. ICD codes were determined and included in the analysis. In cases with more than one ICD code, the high-urgency code was included in the analysis. In the routine examination of the patient in an ambulance by the crew, consciousness (alert, unresponsive, or confused), skin appearance (normal, pale, sweaty, cyanotic, or icteric), visual breathing assessment (normal, superficial, tachypneic, or irregular), pupillary appearance (normal, miotic, mydriatic, or anisocoric) were recorded. In addition, heart rate, systolic, and diastolic blood pressure measurements were recorded. Physical examination evaluations other than pulse, systolic, and diastolic blood pressure measurements were analyzed by classifying them as normal or abnormal.

### 2.2. Statistical analysis

The SPSS 22.0 (IBM Corporation, Armonk, NY, United States) program was used to analyze the data. Descriptive data for categorical variables were expressed as numbers and percentages; Pearson’ chi-squared test was used for comparison. Numerical variables are presented as the mean and standard deviation. Student’s *t* -test was used to compare numerical variables. A binary logistic regression test was used to identify the independent risk factors for ambulance transfer to the hospital, and all possible factors determined as p < 0.20 were included in this analysis. The data were analyzed at a 95% confidence level, and p values less than 0.05 were considered to indicate statistical significance.

## 3. Results 

In 2017 and 2018, 112 emergency number calls were made by a total of 176,104 patients over the age of 65. There was an increase of 31.1% (n = 23,694) in 2018 compared to 2017. An increase of 0.9% was observed in the number of male patients. The mean age of all the included patients was 78.02 ± 8.0 (65–108). Concerning sex distribution, 54.6% of the patients were women. The most common reason for calling an ambulance was medical reasons (Table 1). In 88.6% (n = 156.061) of all cases were calls from the urban area, while 11.4% (n = 20,043) were calls from the countryside.

**Table 1:** Classification of 112 call reasons.

Call reason	Number(n)	Percentage(%)
Medical	157,107	89.2
Other accidents	14,816	8.4
Traffic accidents	2500	1.4
Injury	363	0.2
Suicide	159	0.1
Fire	93	0.1
Work accident	54	0.0
Nonmedical (e.g., health precautions) or unknown	1044	0.5
Total	176,104	100.0

Regarding the call outcomes, it was observed that the outcomes of 3329 patients (1.9%) were not recorded. In 66.2% of the ambulance interventions, the patient was transferred to the hospital (Table 2). When the call outcomes and the call hours were compared, it was seen that during nonworking hours, there was a significantly higher rate of on-site intervention and refusal to transfer than during working hours (p < 0.001).

**Table 2:** Comparison of call outcomes by hours.

Call outcome		Time interval			Total
		08:01–17:00	17:01–24:00	00:01–08:00	
Transfer to hospital	n	57,908	38,486	20,113	116,507
	%	67.0	64.5	66.9	66.2
Transfer between hospitals	n	7883	4608	2143	14,634
	%	9.1	7.7	7.1	8.3
Transfer rejection	n	10,146	8843	3538	22,527
	%	11.7	14.8	11.8	12.8
At-scene intervention	n	4547	3939	2058	10,544
	%	5.3	6.6	6.8	6.0
Other	n	754	339	176	1269
	%	0.9	0.6	0.6	0.7
Exitus (transferred to morgue or left at the scene)	n	2823	1839	1289	5951
	%	3.3	3.1	4.3	3.4
Transfer to home	n	724	474	145	1343
	%	0.8	0.8	0.5	0.8
Total	n	84,785	58,528	29,462	172,775
	%	48.1	33.2	16.7	100

The preliminary diagnosis of the patients was based on the ICD codes. Interventions for which the codes were not identified or the diagnosis codes other than the disease call had been coded were excluded from the analysis (n = 19,834). Based on the remaining data, it was determined that the common codes were for symptom-based or chronic diseases, and the most common diagnosis was cardiovascular disease (Table 3). Trauma (26.9%), infection (31.3%), and psychiatric diseases (27.5%) were more frequent in summer, while respiratory (32.9%), neurological (27.1%), and metabolic diagnoses (26.3%) were more frequent in winter. Furthermore, cardiac arrest (28.8%) events were most frequent in winter, while they were least frequent in autumn (23.2%). A significant seasonal difference was observed (p < 0.001).

**Table 3:** Frequency of diagnosis codes of patients according to systems/problem.

Diagnosis code	Number (n)	Percentage (%)
Other(Symptom-based diagnoses and chronic diseases)	51,984	33.3
Cardiovascular	25,648	16.4
Respiratory	23,593	15.1
Trauma	19,874	12.7
Neurological	10,507	6.7
Cardiac arrest	8008	5.1
Metabolic	5419	3.5
Gastrointestinal	3707	2,4
Psychiatric	3804	2.4
Infection	1548	1.0
Genitourinary	1264	0.8
Intoxication	621	0.4
Gynecological	294	0.2
Total	156,271	100

The records showed that 147,974 patients were evaluated at the scene (cardiac arrest patients, those who were left at the scene due to their death, and those who were transferred between hospitals were excluded), and out of them, 33,835 (22.9%) were left at the scene and 114,139 (77.1%) were transferred to the hospital. The diagnosis codes, sex, event location and time, ICD codes, and physical examination findings (state of consciousness, pupil and respiratory examinations, skin appearances, blood pressures, and pulse) of the patients were found to be significantly associated with their transfer to the hospital (Table 4).

**Table 4:** Relationship between patients’ characteristics and whether they are transferred to the hospital.

Parameter	Subgroup	Intervenedat-scene	Transferred toHospital	p-value
Sex [n (%)]	Female	20,643 (24.9)	62,136 (75.1)	<0.001
	Male	13,191 (20.2)	51,998 (79.8)	
Event location[n (%)]	Rural	3439 (18.6)	15,088 (81.4)	<0.001
	Urban	30,396 (23.5)	99,051 (76.5)	
Time interval[n (%)]	08:00–16:59	15,178 (21.1)	56,793 (78.9)	<0.001
	17:00–23:59	12,947 (25.6)	37,652 (74.4)	
	00:00–07:59	5710 (22.5)	19,694 (77.5)	
Seasons[n (%)]	Winter	8897 (22.8)	30,165 (77.2)	0.640
	Spring	8337 (22.9)	28,094 (77.1)	
	Summer	8542 (25.6)	28,790 (77.1)	
	Autumn	5710 (22.9)	27,090 (77.1)	
ICD code[n (%)]	Other	14,851 (28.6)	37,010 (71.4)	<0.001
	CVS	5372 (21.0)	20,216 (79.0)	
	Respiratory	3159 (13.4)	20,403 (86.6)	
	Trauma	2250 (11.3)	17,582 (88.7)	
	Neurological	1441 (13.7)	9052 (86.3)	
	Metabolic	2646 (48.8)	2772 (51.2)	
	Psychiatric	2269 (59.7)	1532 (40.3)	
	GIS	811 (29.1)	2893 (78.1)	
	Infection	450 (29.1)	1098 (70.9)	
	GUS	364 (28.9)	897 (71.1)	
	Intoxication	98 (16)	516 (84)	
	Gynecological	124 (42.5)	168 (57.5)	
Consciousness[n (%)]	Abnormal	992 (10.9)	8074 (89.1)	<0.001
	Normal	32,843 (23.6)	106,065(76.4)	
Pupil examination[n (%)]	Abnormal	84 (8.6)	894 (91.4)	<0.001
	Normal	33,751 (23)	113,245 (77)	
Respiratory Examination[n (%)]	Abnormal	616 (5.4)	10,727 (94.6)	<0.001
	Normal	33,219 (24.3)	103,412 (75.7)	
Skin[n (%)]	Abnormal	1290 (9.8)	11,891 (90.2)	<0.001
	Normal	32,545 (24.1)	102,248 (75.9)	
Systolic blood pressure*		127.06 ± 25.20	130.89 ± 32.67	<0.001
Diastolic blood pressure*		74.30 ± 13.16	75.57 ± 17.10	<0.001
Pulse rate*		85.55 ± 14.95	90.64 ± 22.52	<0.001

*Mean ± SD

Binary logistic regression analysis of the records of 92,191 patients showed that all the parameters, except for pupil examination (OR: 1.247; %95 CI: 0.942–1.650) and age, were decisive factors for transfer to the hospital. Furthermore, respiratory (OR: 3.215; %95 CI: 2.887–3.580) and skin examination (OR: 2.194; %95 CI: 2.039–2.361) were more closely associated with hospital transfer than pulse and systolic blood pressure (Table 5).

**Table 5:** Binary logistic regression analysis for transferring patients to hospital.

Factor	Subgroup	Odds ratio	95% confidence interval		p-value
			Lower	Upper	
Age		0.998	0.996	1.000	0.076
Sex		1.261	1.219	1.305	<0.001
Hour	08:00–16:59	Reference			
	17:00–23:59	0.780	0.752	0.809	<0.001
	00:00–07:59	0.930	0.887	0.975	0.003
ICD Codes	Other	Reference			
	CVS	1.649	1.576	1.725	<0.001
	Respiratory	2.004	1.892	2.122	<0.001
	Trauma	3.985	3.736	4.250	<0.001
	Neurological	2.780	2.564	3.015	<0.001
Consciousness		1.631	1.491	1.784	<0.001
Pupil		1.247	0.942	1.650	0.123
Respiratory		3.215	2.887	3.580	<0.001
Skin		2.194	2.039	2.361	<0.001
Pulse		1.012	1.011	1.013	<0.001
Systolic blood pressure		1.003	1.003	1.004	<0.001

## 4. Discussion

In the current investigation, a comparison of both years showed there was a 31.1% increment in ambulance interventions for patients aged 65 years and above in 2018 compared to 2017. There was a 5% escalation in the population of patients aged 65 years and over in İzmir in 2017 compared to the previous year [8]. This implies that the increase in ambulance interventions was more than the increase in the number of elderly patients in the population. According to a paper reported by Keskinoglu et al. [6], the number of patients aged 65 years and above who received intervention via an ambulance in 2018 was 6.3-fold higher than that in 2005. The considerable increase that is evident from the regarding paper indicates that several significant arrangements are necessary for EMS planning in this patient group.

In this study, the mean age of patients was 78.02 ± 8.0, and 54.6% of the patients were women. Compared to other studies evaluating emergency department admissions and ambulance calls of elderly patients in our country, the population is older, but the sex distribution is similar [9–11]. In Turkey, in 2019 women constitutes 55.8% of the elderly population and gender life expectancy is longer than men İstatistiklerle Yaşlılar, 2019. Türkiye İstatistik Kurumu, Haber Bülteni (2020). Sayı: 33712 [online]. https://tuikweb.tuik.gov.tr/PreHaberBultenleri.do?id=33712. [accessed 04.12.2020.]. The reason for this difference among sexes may be that we only evaluated the elderly population in this study. Thus, in studies conducted with all age groups in our city, it has been reported that ambulance interventions are more common for male patients [6,12]. 

The organization of emergency health services in rural areas is a global problem. There may be difficulties, especially in delivering trauma cases to major trauma centers in a timely manner [13]. Survival rates of patients with out-of-hospital cardiac arrest or trauma are higher than patients in rural areas [14]. However, ambulance calls are rarely made for these reasons, and inappropriate use of ambulances is common in urban areas where access to a health center is relatively easy [15]. Older age is an independent risk factor for ambulance transport to the ED [16], and the proportion of patients over 65 years old is a predictor of EMS demand in urban areas [17]. In this study, we found that 88.6% of all cases were calls from the urban area. However, we did not compare the rate of using ambulances with the population rate of the elderly by region. A previous study reported that the rural regions had a higher proportion of calls in İzmir [12].

In İzmir, trauma, infection, and psychiatric disease-related ambulance calls for elderly patients were more frequent in summer, while respiratory, neurological, and cardiac arrest were more frequent in winter. Older individuals are more susceptible to changes in air temperature, and the effects of thermal extremes are tended to be larger than other age groups [18]. An increase or decrease in temperature can cause different diseases. A systematic review revealed that temperature reduction increased cardiovascular mortality and respiratory morbidity, and temperature rise increased cardiovascular, respiratory, infectious disease, and heat-related morbidity [19].

In the USA, more than 38% of all patients transported by ambulance are reported to be individuals aged 65 years and older; moreover, it is estimated that approximately half of the patients transported by ambulance will be this group of patients by 2030 [20]. Of all ambulance interventions carried out in İzmir in 2017, 34% of the patients were 65 years and older. Considering the increase in the elderly population of Turkey, it is estimated that the frequency of need for ambulances will increase in patients aged 65 years and older. 

In another research, it is reported that despite 6.5%−9.7% of all ambulance calls are due to falls in individuals aged 65 years and older, only 3.8% of patients are transferred to the hospital in Australia [21,22]. In the same frame of research in the USA, this rate has been reported as 17%, and it is higher in patients over the age of 85 years, those who live in rural areas, and those who live in a nursing home [23]. In our study, we indicate that 12.7% of the calls were made due to traumas, and in most cases, the trauma was caused by accidents other than traffic accidents. Evaluation of the diagnosis codes showed that patients who had injuries due to trauma were transferred to the hospital (OR = 3.98; 95% CI = 3.74−5.45). Thus, the rate of emergency calls due to injuries for elderly patients in Izmir is similar to other countries’ data.

Another interesting finding of our study was that 11.3% of cases with traumatic injuries were intervened on-site and not transferred to the hospital. In previous studies, the percentage of patients who were not transferred to the hospital was reported as 11% to 56% [24]. Elderly individuals are particularly at risk of traumatic injuries, and it seems that the reported rates of intervention via ambulance are similar across many countries. However, emergency teams should be careful when deciding about transfer these patients to the hospital since it is known that these patients often reenter the health system in the next period [25]. Therefore, guidelines are necessary to provide ambulance staff for evaluating traumatic injuries among elderly individuals to help them to decide on transportation to the hospital. Additionally, emergency interventions for elderly patients are different from those for other age groups, as the call rates due to cardiovascular complaints and transport requirements in case of minor problems are higher among the elderly. Finally, these patients also require more advanced life support interventions and longer on-site interventions [26].

Patients aged 65 years and older are likely to suffer from more complicated medical conditions due to comorbidities and medications they continuously use. The undertriage rates for healthcare personnel are higher for traumatic injuries in people over 65 years old than in younger people [27]. In this regard, it is important to transfer patients to a medical institution that is best suited to their medical conditions. In this study, we indicated that 8.3% of all interventions result in transportation to hospitals. The analysis showed that most of these patients were transferred due to cardiovascular-neurological emergencies and traumas. One of the main objectives of the prehospital systems is to transfer patients to the appropriate centers where they could receive definitive treatment, and this is especially important in the case of elderly individuals because of their complicated medical conditions.

Elderly patients are more likely to experience an adverse drug reaction, have a difficult history characterized by cognitive impairments, and suffer from social isolation, abuse, and malnutrition, as well as the atypical appearance of acute coronary syndromes. [28]. Thus, it is important to transport elderly patients to an EMS center that has personnel with experience and equipment that could provide effective care for the elderly. In this study, we did not analyze the hospitals where patients were transported. We recommend studying the factors that affect the transportation of patients to the correct hospital for further investigations. Therefore, we emphasize the importance of identifying regional emergency services and hospitals that will provide the necessary care for these patients.

The current study has several limitations. Firstly, in the analysis of the patients’ diagnoses, the diagnoses made by the emergency medical personnel were evaluated; nevertheless, the definitive diagnoses of the patients were not considered. Health care providers from different educational levels have been working as team leaders in emergency aid Ambulances in Turkey. In this study, the characteristics of the team members who made the recordings were not analyzed. Therefore, there may have been inconsistencies in the physical examination evaluations of the patients. Furthermore, in the regression analysis for identifying factors associated with hospital transfer, only 52.4% of the total patients could be included because data were missing in the remaining cases. Finally, there are differences in prehospital care procedures between countries, so the results of this study should be considered only in the context of Turkey, or specifically, İzmir, as they may not represent other countries or regions.

In conclusion, ambulance interventions for elderly patients in our city are most frequent in urban areas, between 8 AM and 4:59 PM, and during winter. Respiratory pattern, skin examination, state of consciousness, pulse, systolic blood pressure, as well as sex, call time, and the preliminary diagnosis of the ambulance crew, are important factors that affect the EMS crew’s decision to transport an elderly patient to the hospital.
